# FemTech and the equity imperative: *no woman left behind*

**DOI:** 10.3389/frph.2026.1893000

**Published:** 2026-10-27

**Authors:** Nompumelelo Gloria Mfeka-Nkabinde

**Affiliations:** Department of Family Medicine, University of KwaZulu-Natal, Durban, South Africa

**Keywords:** data governance, digital health equity, FemTech, low- and middle-income countries, sexual and reproductive health

## Abstract

FemTech, a rapidly expanding sector of technologies focused on women's health, is an emerging global health megatrend. These technologies impact how menstrual health, fertility, contraception, pregnancy, menopause, and sexual wellbeing are understood and managed. From a health systems perspective, FemTech is best conceptualised as a digitally enabled women's health sector that predominantly operationalises personalised, data-driven care. Most commercial FemTech platforms rely on user-generated data, behavioural analytics, and symptom tracking to individualise recommendations. Using insights from research on young women's sexuality, gendered power relations, and reproductive experiences in low- and middle-income countries, I argue that FemTech can realise its full potential when embedded within equity-driven care models. The paper contrasts the creation and utilisation of FemTech in high-income countries and low- and middle-income countries, and concludes by providing strategies for equity-oriented FemTech.

## 1 FemTech: a global health megatrend and empowerment paradox

Healthcare is increasingly influenced by technological megatrends, among them digital health, artificial intelligence, genomics and data-driven care. FemTech has gained prominence within this landscape as a fast-growing sector addressing gaps in women's health research, innovation and service delivery across high-income countries (HICs), emerging markets and parts of Africa (1–3). The term was coined by the Danish entrepreneur Ida Tin, co-founder of the menstrual and fertility tracking application Clue, and entered scholarly and industry discourse in 2016 as a contraction of *Female* and *Technology* (4). FemTech encompasses mobile applications, wearables, diagnostics, telehealth platforms and data analytics directed at menstrual tracking, fertility prediction, contraception, pregnancy care, menopause management, and sexual and mental health (5, 6). Its ecosystems aggregate population-level data to enable individualised reproductive and sexual health care outside traditional clinical settings (7).

User empowerment is the sector's organising claim. It typically follows when a woman acquires knowledge about her body and its processes, with self-determination as the goal (8). Evidence suggests FemTech use enhances women's self-confidence and intimate experiences (9). In recent years, FemTech has become integral to monitoring pregnancy and foetal development, with advances including virtual care platforms and tools for early detection of pregnancy-related complications, and portable foetal monitoring devices that enable ongoing assessment outside traditional clinical settings (10).

A growing body of critical scholarship cautions that this empowerment claim is paradoxical. Reading across disciplines, Facca et al. (11) argue FemTech's promise of self-knowledge and autonomy coexists with datafication, commercial surveillance and the responsibilisation of women for their own health, frequently within ill-equipped regulatory and legal frameworks. In a thematic analysis of menstrual and fertility application marketing, Hendl and Jansky (8) show how the discourse of empowerment can privilege app-generated “scientific” knowledge over women's own bodily experiences.

Several factors impact FemTech simultaneously: its market scale, the redress of women's historical marginalisation within medical research and health systems, and its socio-political role where medical information, services and rights are restricted. Market analysts estimated the global value of FemTech between US$40 billion and US$66 billion for 2025 and over US$100 billion by 2030 (12–14). FemTech's empowerment potential is not intrinsic to the technology, it is an outcome contingent on the commercial logics, data practices, governance structures and systemic context in which a given tool is embedded. For example, after the Supreme Court overturned *Roe v. Wade*, applications including Flo, Clue and Stardust reported increases in new users, a surge reflecting heightened concern among women seeking to avoid unintended pregnancy under abortion restrictions, and a shifting away from platforms perceived to have weak data protection (15). The same intimate data that makes FemTech valuable renders its users vulnerable to commercial exploitation and legal exposure. Facca et al. (11) frame this as a question of reproductive and menstrual justice, noting that menstrual, fertility and pregnancy data can be repurposed for surveillance or evidentiary use where reproductive rights are contested. The empowerment tool it offers is therefore inseparable from the data-governance regulations in which it operates.

Whether or not one accepts the empowerment narrative, FemTech is structurally driven, globally expanding and system-transformative over the long term. It has evolved from menstrual tracking applications in the mid-2010s into integrated digital ecosystems that shape institutions and markets (10). Shifts including delayed childbearing, declining fertility, increased female labour participation, an ageing female population and a growing awareness of gendered health inequalities, have expanded consumer demand and policy attention (7). Venture capital investment and regulatory attention in HICs signal a long-term trajectory, which advances in wearable biosensors, telemedicine and data analytics further strengthen (16).

## 2 FemTech: innovation without inclusion?

FemTech solutions are not universally accessible and often exclude women from marginalised backgrounds (15). In HICs, FemTech uptake rests on high smartphone penetration, stable connectivity, strong data infrastructure and consumer-driven healthcare markets. In LMICs, particularly in rural settings, digital exclusion remains a significant barrier. Women remain 12% less likely than men to use mobile internet and gender gaps persist in overall mobile and smartphone ownership (17). Device sharing and prohibitive data costs are therefore ordinary conditions of women's lives, and FemTech built for uninterrupted connectivity and individual use risks excluding those most in need. Structurally, women's reproductive choices are constrained by poverty, gender inequality and limited access to quality services. Research from rural KwaZulu-Natal shows how young women's sexual and reproductive lives are influenced by coercive relationships, transactional sex, inadequate sexual communication in schools and families, and moralistic health care provider attitudes (18–22). Across parts of sub-Saharan Africa, India and South Asia, menstruating girls and women may face restrictions on cooking, schooling, religious participation or interaction with men, on the grounds that menstruation renders them unclean (23). Unmet menstrual health needs intersect with poverty, education, gender equality, and sexual and reproductive health, which are all relevant to make progress on the Sustainable Development Goals (24). FemTech that meaningfully serves such settings must engage these structural realities.

Corbin (6) further contends FemTech in HICs is predominantly funded by men, which may embed assumptions about women's needs while reinforcing dominant gender inequalities. Critical scholarship identifies a deeper danger: that commercial FemTech entrenches a datafied, market-driven model of women's health under the language of empowerment, blurring the line between healthcare and consumer data extraction (11). These dynamics are not confined to high-income markets. Studies of FemTech in emerging economies describe reproductive and bodily data becoming objects of surveillance and monetisation, raising the prospect that women in LMICs are incorporated into FemTech ecosystems as sources of data and not as beneficiaries of equitable care (25). A systematic review of 112 FemTech applications in China found that most defined women chiefly as mothers, overstated the reliability of fertility and contraception claims, and employed extractive data practices, with a third mandating data collection as a condition of use and the majority sharing data with third parties (26). Chen advances the concept of *patriarchal digitality* to describe how digital tools can automate existing gender hierarchies. Where regulatory protection, digital literacy and bargaining power are weakest, the distance between FemTech's empowerment rhetoric and its material effects is widest.

## 3 Equity-driven FemTech

In this manuscript, I understand equity as the fair distribution of benefit derived from a technology, and the narrowing of unjust gaps in health information, services and outcomes between women in different social positions. Four dimensions are useful to locate a tool on the equity spectrum: 1) funding models, whether venture-backed and direct-to-consumer, employer- or insurer-funded, or public and donor-financed; 2) primary purpose, whether consumer self-tracking or population health, with clinical care capable of sitting at either axis depending on how it is funded, governed and delivered; 3) data models, whether extractive or minimising; and 4) mode of delivery, whether high-bandwidth application or wearable, or low-bandwidth voice, SMS or USSD. These axes determine where a tool sits (Figure 1).

**Figure 1:**
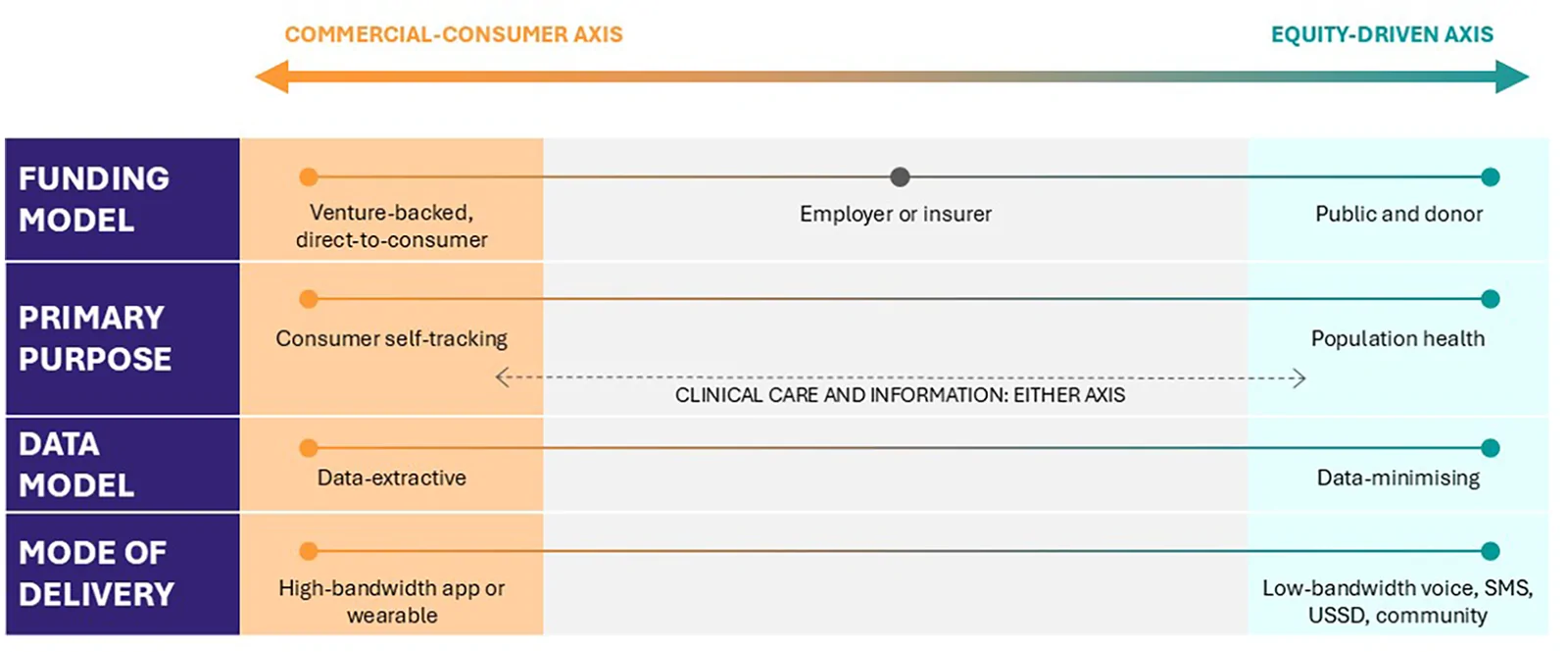
Locating a FemTech tool on the equity spectrum.

An equity-driven tool can further be assessed against explicit criteria. Is it designed with the women facing the steepest access constraints in mind? Does it default to the lowest-barrier channel? Can it be embedded within a health system? Does it protect the data it collects? Is it financed sustainably? These questions underpin the strategies proposed in Section 4. Programmes developed in India and Africa have largely answered the first two questions by prioritising low-bandwidth, population-level interventions: SMS, voice platforms and basic applications with offline functionality or minimal data cost. In Mumbai's low-income settlements, the mMitra voice-message programme improved maternal health knowledge and practices, an intentional design choice for contexts of low literacy and intermittent data access (27). India's Kilkari programme demonstrates that such reach is achievable at national scale through public-system integration (28).

South Africa's MomConnect programme is an illustration of how a government-backed, basic-messaging application can be embedded into routine maternal services. It is delivered on basic phones at no cost to the user, routed through the national health system serving 80% of the population, and coupled to a helpdesk that channels women back into routine antenatal services (29). The equity gain is identifiable: women who cannot pay subscriptions for commercial platforms are reached and connected to care. Given South Africa's social and health system inequalities, the same messaging technology sold direct-to-consumer would not produce that gain, which is precisely the argument this paper advances. Evidence from elsewhere supports the point. Targeted SMS increased postpartum care-seeking and family planning uptake in peri-urban Kenya (30), while Rwanda's RapidSMS experience showed that improvements in service utilisation were strongest where the platform was paired with training, supervision and equipment as broader health-system investments (31).

While the above approaches are equity-oriented, they have limitations. The first is technological depth. Many programmes in lower-resource settings offer health education, appointment reminders and behavioural nudges, without physiological monitoring, hormonal diagnostics, biomarker-driven precision medicine or AI-enabled risk prediction (32). The second is clinical integration. Digital maternal and reproductive health tools frequently operate alongside routine services without being embedded in electronic health records, health information systems are fragmented, and data flows between community platforms and facilities are inconsistent (33). Thirdly, scaling depends on donor funding or public budgets that are vulnerable to fiscal constraint and shifting political priorities, and reviews of mHealth in antenatal care across sub-Saharan Africa identify these systemic and financing barriers to scale (34). The strategic task is therefore to add clinical depth and financial durability to equity-driven platforms without compromising access. A tiered model resolves the apparent tension: a low-barrier channel of voice, SMS or USSD remains the universal baseline, and higher-technology clinical functionality is layered on where infrastructure, connectivity and health-system readiness allow, so that sophistication never becomes a precondition for access.

## 4 Strategies for an equitable FemTech future

### 4.1 Prioritise low-technology design

Equity-oriented design defaults to the lowest-barrier channel rather than to technological sophistication. Given persistent gender gaps in mobile internet use, smartphone ownership and mobile money (17), voice, SMS and USSD reach women who share devices, hold intermittent airtime and cannot assume connectivity.

### 4.2 Support women-led innovation and co-design

Backing women founders, particularly in LMICs, diversifies product priorities and counters an industry funded predominantly by men that “makes assumptions about women's needs and desires that may not necessarily align with reality” (6). Male dominance is woven into the infrastructure, leadership and algorithms of digital health, so that gender-specific constraints are ignored under an assumption of gender neutrality (35). Equity-centred design offers the corrective, incorporating the real-life experience of end users as co-designers throughout so that inclusion is not a checklist (36, 37). Given high levels of gender-based violence in many LMICs, safeguarding belongs in the product requirements: safe messaging defaults, quick-exit functions, consent flows, risk-aware notifications and referral pathways.

### 4.3 Integrate FemTech into health systems

Integration into workflows, health information systems and referral pathways determines whether a tool informs clinical decisions or remains a parallel layer. At primary care level, co-design with nurses, community health workers and clinic managers should reduce workload through triage prompts, appointment reminders, defaulter tracing and referral, rather than generating new administration. Integration also means alignment with national digital health strategies and universal health coverage, adopting open data standards and building interoperable, reusable systems that connect community platforms to facilities (38).

### 4.4 Secure sustainable financing

The WHO Science Council argues that digital health infrastructure in LMICs should be viewed “not as a cost but an essential strategic investment” (39), and the WHO Global Strategy calls on countries to identify sustainable financing models (38). Positioning FemTech within women's health and maternal and child health programmes, and framing it against the Sustainable Development Goals can strengthen the investment case since menstrual and reproductive health cuts across goals on health, gender equality, education, and poverty (24). Practically, developers and investors can cross-subsidise low-technology versions from high-technology products, public budgets and global health funders can sustain system integration through service-level agreements tied to coverage and quality indicators, insurers and employers can fund tools for insured populations, and partnerships with mobile network operators can zero-rate data for essential women's health services.

### 4.5 Govern data and confront algorithmic bias

Data protection warrants treatment as a strategy in its own right. The safeguarding of data is regulation as much as design: enforceable data-protection law, purpose limitation, restrictions on third-party sharing, and oversight of cross-border data flows. South Africa's Protection of Personal Information Act offers a starting framework, though enforcement capacity and FemTech-specific guidance remain limited, and many tools used locally are governed by the laws of the countries in which their developers are based. Representativeness is the second, less-discussed equity issue. Algorithm-driven FemTech is only as sound as its training data; models built predominantly on women in high-income settings may mislead or incorrectly predict for the populations this paper concerns. Equity requires that training data include women from LMIC and rural settings and that tool performance be validated in those populations before deployment.

## 5 Conclusion

FemTech is a global health megatrend impacting women's health and healthcare markets. It emerges at the intersection of digital transformation, demographic transition and gender justice advocacy. Yet, its expansion does not guarantee equity, and its promise of empowerment is not automatic. As critical scholarship reminds us, FemTech can extend women's health agency and entrench commercial surveillance simultaneously (11). The WHO frames digital health as a route to universal health coverage by widening the population able to reach services (38); whether FemTech serves that end depends on the choices made about who it is designed for. Without deliberate integration into health systems, inclusive design, robust data protection and sustainable financing, FemTech may reproduce the very inequalities it claims to redress. Equity is not a by-product of innovation; it is a design and policy decision that must be resourced. The critical question for health care leaders is no longer whether FemTech will grow; it is whether that growth will expand justice, autonomy and health access for women across diverse social contexts.
